# Eosinophils in obesity and obesity-associated disorders

**DOI:** 10.1093/discim/kyad022

**Published:** 2023-11-14

**Authors:** Yanan Hu, Svetoslav Chakarov

**Affiliations:** Shanghai Institute of Immunology, Shanghai JiaoTong University School of Medicine, 280 South Chongqing Road, Shanghai, China; Shanghai Institute of Immunology, Shanghai JiaoTong University School of Medicine, 280 South Chongqing Road, Shanghai, China

**Keywords:** eosinophil, adipose tissue, obesity, immune metabolism

## Abstract

Despite the rising prevalence and costs for the society, obesity etiology, and its precise cellular and molecular mechanisms are still insufficiently understood. The excessive accumulation of fat by adipocytes plays a key role in obesity progression and has many repercussions on total body physiology. In recent years the immune system as a gatekeeper of adipose tissue homeostasis has been evidenced and has become a focal point of research. Herein we focus on eosinophils, an important component of type 2 immunity, assuming fundamental, yet ill-defined, roles in the genesis, and progression of obesity and related metabolic disorders. We summarize eosinophilopoiesis and eosinophils recruitment into adipose tissue and discuss how the adipose tissue environments shape their function and vice versa. Finally, we also detail how obesity transforms the local eosinophil niche. Understanding eosinophil crosstalk with the diverse cell types within the adipose tissue environment will allow us to framework the therapeutic potential of eosinophils in obesity.

## Introduction

Obesity has emerged as a global epidemic, with occurrence almost tripling since 1975. Nearly 2 billion people (1 in 4 humans worldwide) are overweight [1], consecutively leading to a pandemic in obesity-related diseases such as type 2 diabetes (T2D) [2, 3], cardiovascular disease [4], non-alcoholic fatty liver disease (NAFLD) [5], non-alcoholic steatohepatitis (NASH) [6], and cirrhosis and cancer [7]. The metabolic perturbations caused by obesity have led to the frame of this pathology as a metabolic disease. More recently, obesity and related disorders have been associated with low-grade chronic inflammation also called “meta-inflammation” and are starting to be considered inflammatory diseases driven by metabolic dysregulation [8–11]. Linking such disorders to inflammation implies that their causes require reconsideration and an urgent need for a better understanding of the underlying biological pathways.

Inflammation is an immune response triggered by a wide variety of stimuli and involves the coordinated action of different immune cells [12]. Among these cells, eosinophils play a crucial role in host defence and tissue remodelling by producing a large variety of substances including cytotoxic and lipid mediators, chemokines, and cytokines [13–16]. During inflammation, eosinophils migrate into the target tissue, becomes activated, and release products that further exacerbate the local inflammation. In some cases, this causes tissue damage, but also promotes tissue remodelling and repair [13–18].

Eosinophils were first described by Paul Ehrlich at the end of the 19th century. They were defined by their capacity to be stained by acidophilic dyes (such as eosin) and known to be involved in allergic asthma. Nowadays, eosinophils are most known as highly specialized effector cells recruited to the tissues as a result of T helper type 2 (Th2) immune responses [19]. From an evolutionary point of view, eosinophils are primitive myeloid cells maintained in vertebrates, including reptiles and fishes [20], suggesting their beneficial role in protective Th2 immunity against parasites [13]. Aside from their anti-microbial and innate immune functions, eosinophils are present at steady-state in certain organs, and their homeostatic role in regulating local immunity and/or remodelling/repair, or so-called “LIAR” hypothesis has been recently proposed [21]. In agreement, eosinophils were found to play a role in tissue morphogenesis and homeostasis for example during reproduction [22], pregnancy [23], mammary gland development [24], maintaining of epithelial barrier integrity [25], as well as metabolic homeostasis [26], glucose tolerance during obesity [27], and adipose tissue (AT) fitness in aging [28]. In addition, the LIAR hypothesis states that eosinophil activities are largely influenced by the tissue microenvironment [21], similar to other immune cells such as macrophages and their tissue-specific niche of residency [29]. Eosinophils can integrate cues from the tissue-shaping their tissue specific function. This is supported by evidence that tissue-resident eosinophils exhibit distinct properties from mature bone marrow (BM) or blood cells [19, 30].

As a main energy storage in the body, AT quickly responds to both caloric deprivation and excess [31]. However, these responses place massive demands on AT to dynamically adapt to the changing nutrient environment, a process generally referred to as adipose tissue remodelling. The regulation of energy uptake and release driven by adipocytes are well understood at the molecular and cellular levels [32]. These actions require a supporting network of non-adipocyte cells, and in recent years eosinophils emerged as key regulators of AT physiology. Accordingly, the connection between obesity and eosinophils was recently proposed [33]. Here, we further discuss the uniqueness of AT eosinophils (ATE), their changes in high-fat diet (HFD)-induced obesity model, their interactions with other cells in adipose tissue microenvironment, and the important role of eosinophils in obesity and related metabolic complications.

## Eosinophilopoiesis and adipose tissue homing

### Eosinophilopoiesis

**Figure 1:**
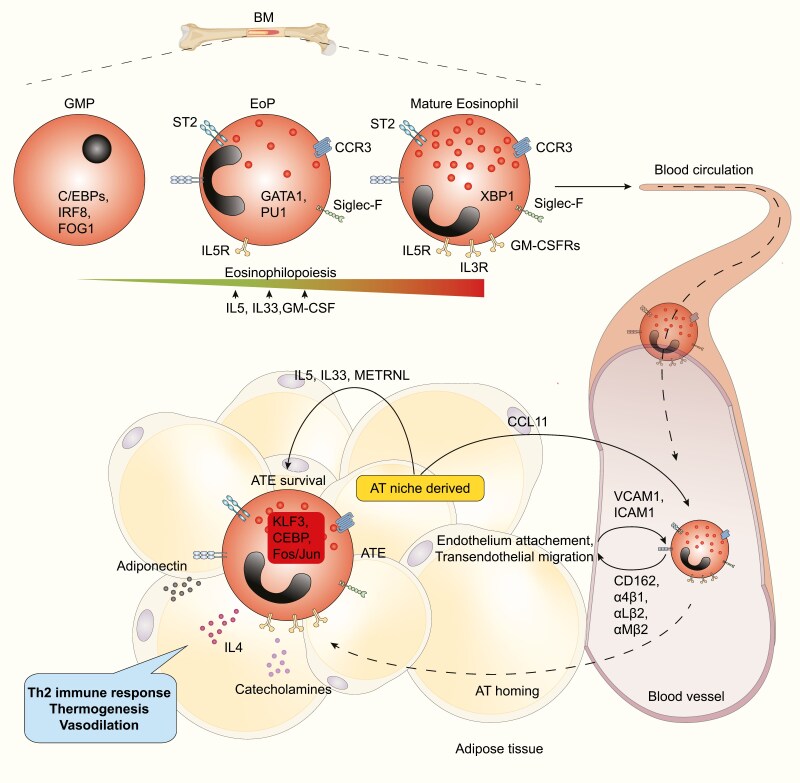
adipose tissue eosinophils differentiation and recruitment. Eosinophils develop in the bone marrow (BM), from granulocyte/macrophage progenitors (GMP), to mature eosinophils passing by eosinophil-lineage-committed progenitors (EoP) stage. This transition implies the expression of various transcriptional factors such as CEBPs GATA1 and XBP1 and under the control of cytokines such as IL5, IL33, and GM-CSF. Eosinophils exit the BM into peripheral blood as mature cells expressing Siglec-F, CCR3, and ST2. Recruitment of eosinophils in adipose tissue (AT) depends on CCL11 secretion by AT niche. The extravasation of eosinophils depends on their interaction with endothelial cells via molecules such as ICAM1, VICAM1 expressed by endothelium and CD162, α4β1, αLβ2, αMβ2 on eosinophils. In the AT, AT-derived factors promote AT eosinophils (ATE) reprograming and subsequent ATE-specific function.

In mice, eosinophils originate in the bone marrow (BM) from granulocyte/macrophage progenitor (GMP). Subsequently, GMP gives rise to eosinophil lineage-committed progenitor (EoPs) that terminally differentiate into mature eosinophils. The decisive steps orchestrating eosinophilopoiesis are driven by a complex interplay of lineage-determining transcription factors (TF), including GATA-binding protein (GATA)-1 and -2 [34–37], CCAAT/enhancer-binding protein (C/EBP) members [38–40], ETS family member PU.1 [41–43], IFN response factor-8 (IRF8) [44], and X-box-binding protein-1 (XBP1) [45]. GATA-1 was reported as the most selective TF driving the maturation of eosinophils from EoPs. Accordingly, genetically modified mice in GATA1 expression (ΔdblGATA mice) specifically lack circulating and tissue-resident eosinophils [37] (Figure 1). In addition to GATA1 upregulation, eosinophil maturation in BM is associated with an increase of interleukin (IL) 5 receptor α (IL5Rα) expression—the receptor for eosinophils primary growth factor—IL5 [46]. Indeed, IL5Rα acquisition was reported as a critical event in EoP maturation and expansion [46–48]. Later, in mature eosinophils, IL5 initiates their release from BM, and tissue-derived IL5 enhances their tissue-specific survival [22, 26, 49, 50]. However, IL5 is not completely indispensable at steady-state, as circulating eosinophils were only slightly reduced in IL5-deficient mice [46, 49], suggesting that other factors could drive eosinophil lineage commitment. Such factors include Colony Stimulating Factor 2 Receptor (CSF2R) β-binding cytokine IL3 and granulocyte-macrophage colony-stimulating factor (GM-CSF), which have been shown partially dispensable for steady-state eosinophil differentiation [51, 52]. More recently it was demonstrated that the alarmin IL33 could directly interact with EoPs through their IL1 receptor-like 1 (IL1RL1 or ST2) expression, driving both their expansion and increase of their IL5Rα expression, thereby regulating eosinophil homeostasis [53–56]. In addition, aging-, obesity-, inflammation- or allergy-induced IL33 fluctuation could directly target BM EoPs and/or tissue eosinophils and modulate their number and activation [57–61] (Figure 1).

While the TF and cytokines described above are equally important for mouse and human eosinophils differentiation in BM [62], it appears that human GMP do not give rise to eosinophils. Rather human eosinophils arise from an L5Rα^+^ common myeloid progenitor (CMP) [63].

At the end of eosinophilopoiesis, fully mature eosinophils expressing surface markers including IL5Rα, C-C chemokine receptor 3 (CCR3), siglec-F in mice or siglec-8 in humans are released into the circulation [64–66]. Under homeostatic conditions, blood eosinophils account for less than 5% of total circulating leukocytes with a relatively short half-life (3–18 h) before they migrate to the tissue [67], where their survival was estimated to be 2-5 days [68]. The events leading to eosinophils entry in any extravascular compartment involve leukocyte and endothelial adhesion molecules. Initially, eosinophils are attached to the activated endothelium through their expression of P-selectin ligand CD162 and P-selectin on the endothelium (39, 40). The subsequent transendothelial migration is mediated by eosinophil expression of integrins such as α4β1 (CD49d/CD29), αLβ2 (CD11a/CD18), and αMβ2 (CD11b/CD18) interacting with endothelium vascular cell adhesion molecule 1 (VCAM-1) and intercellular adhesion molecule (ICAM-1).

### ATE homing

At steady state, ATE constitute around 4% of AT stromal/vascular fraction (SVF) [27], and as for most organs, the axis CCR3-eotaxins plays a crucial role in eosinophil’s chemotaxis. The eotaxin family comprises three members—eotaxin1 (CCL11), eotaxin2 (CCL24), and eotaxin3 (CCL26)—with tissue-specific expression patterns [69, 70]. Eotaxins can be produced by a variety of immune and non-immune cells including but not restricted to fibroblast, smooth muscle cells, endothelial cells, epithelial cells, macrophages, lymphocytes, and eosinophils themselves [70]. In AT, it was shown that CCL11 is produced by multipotent stromal cells (MSC)/adipocyte progenitors (AP), and mature adipocytes [19, 71–74]. Production of CCL11 by MSC was dependent on MSC/Group-2 innate lymphoid cells (ILC)2 interaction and IL4/IL13 production [71]. In addition, the ILC2-derived IL5 supported ATE survival and sustained their IL4 secretion in epididymal white AT (eWAT) [26, 71]. CCL11 was also released by mature adipocytes, in response to autocrine axis fibroblast growth factor 21 (FGF21)/ co-receptor β-Klotho (KLB) induced by cold exposure [73, 75], thereby promoting eosinophil recruitment in subcutaneous WAT (scWAT) [73]. Other molecules such as α L- and α 4-mediated integrin and the cross-talk between IL-5 receptor and CD300f also affect ATE accumulation [27, 76].

Upon infiltrating the AT, eosinophils undergo phenotypic and functional specialization driven by the AT niche itself, promoting expression of TF such as Kruppel-like Factor 3 (KLF3), CEBPs, Fos/Jun in eosinophils [19, 30, 77]. As a consequence, the eosinophil-derived Th2 cytokines IL4 and IL13 orchestrate scWAT browning and brown adipose tissue (BAT) thermogenesis [73, 76, 78] (Figure 1).

In murine models of obesity, a negative correlation between weight gain and ATE abundance were observed [26, 27, 57, 79–81]. This was the result of the decrease of AT-derived chemokine CCL11 and cytokine IL33 with the HFD feeding [57, 79]. Accordingly, mice overexpressing CCL11 in AT, or the administration of IL33 restored ATE fraction and suppressed obesity [57, 79]. However, there is currently controversy regarding ATE abundance during obesity discussed above. Beyond the differences in experimental approaches, conditions, and species that could influence such contradictory results, a further question is raised—Do functionally and phenotypically distinct ATE subpopulations inhabit the AT?

Indeed, eosinophil heterogeneity has been reported in other tissues. In the lung, SiglecF^int^CD62L^+^CD101^lo^ resident eosinophils (rEos) were present at a steady state, whereas allergic inflammation promoted the recruitment of SiglecF^hi^CD62L^-^CD101^hi^ inflammatory eosinophils (iEos) in human and mice [50]. Mice lacking rEos showed increased Th2 immune response after allergic challenges [50]. In the gut, using single-cell RNA sequencing (scRNAseq) technology, two independent studies identified eosinophil’s heterogeneity [59, 82]. According to Gurtner *et al.* ATE can be characterized as “active” and “basal” subpopulations [59]. The “active”—expressing CD80 and programmed death-ligand 1 (PD-L1)^—^were endowed with antibacterial and regulatory functions and were found in specific subtissular niches [59]. More recently, Li *et al.* characterized a subpopulation of neuromedin U receptor 1 (NMUR1)^+^ intestinal eosinophils implicated in both goblet cell differentiation and mucosal immunity against helminth [82]. Finally, AT transcriptome analysis identified AT-specific eosinophils reprogramming. Compared to blood eosinophils, ATE were endowed with tissue-specific TF activity such as KLF, CEBP, and Fos/Jun family members [19, 30]. However, an updated understanding of ATE heterogeneity is still lacking and a thorough characterization is urgently needed. Nevertheless, it is currently accepted in the field that the presence of eosinophils within AT provides protection against obesity, promotes thermogenic energy expenditure and metabolic homeostasis in mice [83, 84], and through mechanisms discussed below.

## Role of ATE in obesity

During obesity, when energy intake consistently exceeds energy expenditure, the adipose tissue expands via increase in adipocyte cell size (hypertrophy), and recruitment of AP (hyperplasia) [32]. In recent years an accumulation of pro-inflammatory immune cells in WAT during obesity [85, 86] has been evidenced, and progressively the immune system as a key regulator of AT homeostasis become clearer [87]. Pro-inflammatory, type 1 immune response (Th1) is associated with AT dysfunction in obesity [11, 88], on the other hand, Th2 immunity drives AT homeostasis and repair [83]. Wu *et al* first reported a protective role of eosinophils against obesity in mice [27]. The authors showed that the absence of ATE in ΔdblGATA mice on HFD promoted weight gain, impaired glucose tolerance, and decreased insulin sensitivity compared to wild-type (WT) animals. In contrast, increased Th2 immunity and eosinophilia of WT mice during HFD feeding improved obesity-associated complications [27]. Following the trend, other studies confirmed the finding. Overall, increased eosinophilia and Th2 immune response, using whole body parasite infection or the overexpression of IL5 prevented weight gain, decreased adipocyte size, and improved glucose intake, and insulin sensitivity.

The mechanism underlying the protective driven by eosinophil’s role was reported to be dependent on increased thermogenesis [73, 75, 77, 78, 89]. Thermogenesis is the metabolic expenditure of energy as heat and is the primary way in which mammals lose dietary-derived energy. Indeed, while WAT stores excess energy as triglycerides, BAT produces heat in cold conditions, and plays a pivotal role in maintaining body temperature. In addition to brown adipocytes, beige adipocytes—a distinct cell type that regulates thermogenesis *in vivo*—have also been identified [90]. Both brown and beige adipocytes express uncoupling protein 1 (UCP1), which allows them to decouple mitochondrial respiration from ATP synthesis and dissipate energy as heat. Beige adipocytes differentiation can be induced within WAT in response to certain stimuli such as cold exposure, or β-adrenergic receptor (AR) activation, and Th2 cytokines. This phenomenon is referred to as “WAT browning”, or “beiging”. Both, brown and beige AT were associated with obesity protection in rodents and was discussed elsewhere [91].

Indeed, the main mechanism eosinophils promote thermogenesis is through Th2 cytokine secretion such as IL4/IL13, and signal transducer and activator of transcription (STAT) 6 signalling downstream IL4Rα activation [78]. IL4 increased insulin sensitivity in mice fed on HFD, reduced their weight gain, and induced AT expansion [92, 93]. Inversely, lack of IL4/IL13, IL4Rα, or STAT6 signalling reduced insulin sensitivity in mouse models of diet-induced obesity, decreased beige adipocyte formation, cold-induced thermogenesis, and decreased energy expenditure [93, 94].

The data summarized above suggest that eosinophils promote leanness, at least in mice. However, these studies use mice models in which eosinophils are absent or in excess. Although eosinophils are present in lean, and reduced in obese mouse AT, Bolus *et al*. noted that elevating ATE to the physiological level during obesity did not improve the metabolic health described by others [81]. Furthermore, elevating eosinophils with acute helminth antigen treatments, did not protect against diet-induced obesity [79]. Finally, adipocyte-derived CCL11 drove accumulation of ATE with the HFD, thereby promoting adipocyte maturation, lipid accumulation, and glucose intolerance [74].

In humans, the data regarding the role of eosinophils in AT homeostasis, and metabolic health are more complicated and largely unknown. Studies reported a positive correlation between blood eosinophilia and body mass index (BMI) [95–97], and elevated human ATE count was associated with metabolic syndrome [98]. In addition, anti-IL5 therapy, depleting eosinophils, significantly decreases BMI in patients with severe asthma [99]. On the other hand, human high blood eosinophilia was associated with a decreased risk of T2D [100]. Accordingly, increased eosinophil count caused by non-pathogenic parasite infection correlates with reduced T2D and associated metabolic disorders in humans [101].

In view of these contradictory results, addressing the role of eosinophils in human AT is urgently needed, and eosinophil-based treatment for metabolic disorders remains a theory. Nevertheless, evidence points out that the accumulation of eosinophils in AT supports metabolic health, and we are going to further discuss the actor implicated in ATE recruitment, survival, and activation in the next part of the review.

## AT niche/eosinophil cross-talk

WAT is endowed with well-organized stroma, nerve bundles, and immune cell network. The main tissue mass is composed of adipocytes; however, they only represent around 20% of the total cellularity of eWAT [102, 103]. The remaining 80% of the tissue includes fibroblasts, vascular cells, immune cells, stromal cells, and neurons [102–104], each contributing to WAT remodelling in response to nutrient excess or deficiency and potentially affecting ATE recruitment and activation. In this part, we will overview how potential cell components of the AT niche dictate ATE function and vice versa.

### Type 2 innate lymphoid cells

In adult, resident ILC2s are derived during different phases of development. Fetal and neonatal ILC2 first seed the tissues during embryogenesis, and the first days after birth respectively. Later in adult, embryonic-derived ILC2 are slowly replaced by BM-derived cells [105]. The role of ILC2 in metabolic homeostasis has been reported in both humans and mice, directly through the production of enkephalin peptides and promoting beiging [106] or/and indirectly via mechanisms involving cross-talk with ATE [107]. Indeed, AT ILC2 secrete IL5, IL13, and IL33 thereby promoting ATE recruitment and survival [26, 53, 94]. IL33 also drives the recruitment and/or proliferation of ILC2 themselves. However, the cellular origin of IL33 and the mechanisms leading to its secretion remain poorly understood. In literature, it was reported that a variety of AT cell types could produce IL33 at steady state, including podoplanin (Gp38)^+^ stromal cells of fat-associated lymphoid clusters, Gp38^+^ fibroblasts, mesenchymal cells, leptin receptor (LepR)^+^ Sympathetic Associated Perineurial Cell (SAPC), adventitial stromal cells (ASC), or endothelial cells [55, 56, 108–112]. Moreover, IL33 secreting ASC were identified as ILC2s subtissular niche, and ASC-derived thymic stromal lymphopoietin (TSLP) drove ILC2 accumulation and expansion, thereby promoting Th2 immune response [56]. Eosinophils could also produce IL33, however, the role of ATE-derived IL33 on ILC2 was not assessed [77]. The IL33 signal is also required for ILC2 derived IL5 secretion, and ILC2s are key producers of systematic and AT local IL5 required for homeostatic ATE recruitment and maintenance [26, 113]. ILC2-dependent ATE activation during acute cold exposure and subsequent IL-4/IL13 secretion have been reported to be important for AT browning and thermogenesis. In regards to these examples, not surprisingly, eosinophils tightly follow ILC2’s fluctuation.

Indeed, obesity-induced meta-inflammation is associated with systematic and AT local up-regulation of pro-inflammatory mediators such as interferon γ (IFNγ), tumour necrosis factor α (TNFα), IL6, or IL1β [11, 114, 115]. In turn, these mediators significantly reduce ILC2s abundance in AT through decrease in their proliferation and tampering their functions [108, 116]. Subsequently, obesity-induced meta-inflammation leads to a decrease in eosinophil, thus promoting pathological AT expansion and insulin resistance [108, 116]. Inversely, the adoptive transfer of activated ILC2s into obese mice promotes eosinophilia and reduces weight gain and glucose intolerance [117]. The underlying mechanisms involve IL4/IL13 secretion by ATE and AT macrophage (ATM) activation discussed below.

### Macrophage

**Figure 2:**
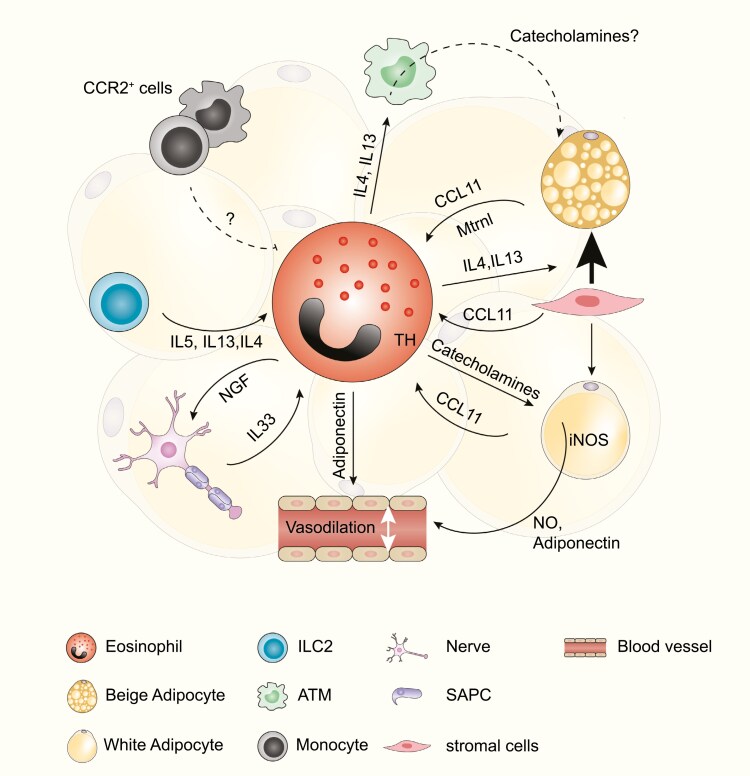
ATE niche. Different AT cell components will provide distinct signals that will dictate ATE specific functions. Inversely ATE will provide signals controlling the AT environment.

Among AT immune cells, ATM represents 5–10% of SVF in lean, steady-state conditions [86], and plays a critical role in AT homeostasis, discussed elsewhere [88]. It was first reported that Th2 immune response and IL4 promote tissue macrophage proliferation [118, 119]. Subsequently, a decade ago, Wu *et al.* were the first to report eosinophils in lean, healthy AT, that could promote the so-called “macrophage polarization” toward an anti-inflammatory phenotype, through an IL4/IL13 dependent process [27]. Interestingly, age-related decline of ATE was observed, correlating with the switch from Th2 to Th1 AT environment and pro-inflammatory ATM phenotype [27, 28]. Restoring ATE distribution by adoptive transfer of eosinophils from young mice into aged recipients dampens age-related meta-inflammation [28]. In contrast, both eosinophil depletion and IL4/IL13-IL4Rα axis ablation have been linked to impaired thermogenesis and beiging as a result of loss of ATM-derived anti-inflammatory mediators [78]. It was also proposed that anti-inflammatory ATM phenotype, induced by cold exposure, could promote thermogenesis by tyrosine hydroxylase (TH)/ norepinephrine (NE) pathways downstream of IL4Rα-STAT6 pathway [78, 120]. However, the main contribution of macrophages to AT energy expenditure through their production of catecholamines is currently under debate. Several research groups described that rather than synthesizing catecholamines, macrophages are instead implicated in catecholamine uptake and degradation [121, 122]. In addition, IL4 did not affect ATM catecholamine secretion *in vivo*, and ATM did not express a significant amount of TH, the machinery necessary for NE synthesis [123]. Following the trend more studies provided opposite evidence about macrophage polarization and TH/NE axis [124, 125]. Thus, additional research is needed to clarify the relationship between ATE/ATM/catecholamines secretion and AT thermogenesis and beiging (Figure 2).

Obesity induces a drastic increase in ATM number, reaching up to 40–50% of the SVF in mice [86] and humans [126]. Subsequently, ATMs sustain obesity-associated meta-inflammation as they are the main source of pro-inflammatory mediators in obese fat [11, 88, 127]. As consequence, ATM accumulation leads to a drastic decrease in AT ILC2 and ATE, thus promoting obesity and its complications [108, 116]. It was proposed that obesity-associated ATMs were of BM origin [86]. In addition, mice deficient in C–C chemokine receptor type 2 (*Ccr2*)^−/ −^, a chemokine receptor required for monocytes to egress from BM, are protected from obesity-associated complications but not weight gain [128]. The recruited monocyte-derived ATMs are often found forming crow-like structures (CLS), in which they act as debris and lipid-droplet scavengers [129, 130]. CLS are suggested to preserve tissue integrity in the face of massive adipocyte cell death [131]. Interestingly, ATE accumulation during obesity were dependent of a population of CCR2-expressing cell [132]. Accordingly, *Ccr2*^–/–^ mice increased ATE with the HFD feeding which led to increased AT Th2 immune response [132]. This was a result of an increase of AT derived IL5 in *Ccr2*^–/–^ mice. Finally, the recruited ATE in *Ccr2*^–/–^ were located in close contact with dying adipocyte within the CLS [132]. On the other hand, it was reported that macrophages can produce CCL11 and promote eosinophil recruitment [133].

Despite the fact that the examples cited above provide cardinal foundations to understand ATE/ATM interaction in metabolic diseases such as obesity, they had limitations stemming from their use of the outdated “M1/M2” macrophage classification proposed more than 20 years ago [134]. According to this model, Th2-related “M2” ATM are anti-inflammatory and are the dominant population in lean fat [127]. In contrast, Th1-related or pro-inflammatory—“M1” ATM was thought to be induced during obesity and to be associated with tissue damage [127, 135]. As such, the controversial results cited above are not surprising given that the M1/M2 polarizations represent only the extremes of a full spectrum of macrophage activation states [136, 137]. Indeed, ATM are heterogenous population of cells with diverse origins and each ATM population is endowed with specific functions [88, 138–142]. Thus, depicting the precise role of each ATM subpopulation on ATE recruitment/function and vice versa is the next challenge toward understanding metabolic diseases.

Nevertheless, these combined studies suggest that the “Ménage à trois”—ILC2/ATE/ATM—could contribute to AT metabolic health through the regulation of AT inflammation, thermogenesis, and insulin resistance.

### Adipocytes and adipocyte progenitors

Approximately 10% of adipocytes are renewed annually by a continuous turnover from MSC/AP. AP are a heterogeneous population of cells giving rise to the different lineage of adipocytes (for review [143]). Interestingly, ATE were able to directly trigger adipocyte maturation or induced AP dedifferentiation into beige adipocytes [94]. Indeed, ATE-derived IL4 were reported to stimulate the proliferation of adipocyte progenitors in scWAT and increased their expression of beige markers such as CD137 and TMEM26 [90, 94]. Interestingly, a population of platelet-derived growth factor receptor α (PDGFRα)^+^ AP expression IL4Rα were responsible for the effects of IL4 on the AT beiging, and via downstream mechanisms involving STAT6 activation [94]. Accordingly, adipocyte-derived FGF21 promoted WAT beiging through CCL11-dependent ATE recruitment and subsequent IL4 increased in WAT [73]. The effect was partially blocked using antibody against CCL11, implying the pivotal role of eosinophils in this process. IL4 could also inhibits adipogenesis by downregulating the expression of peroxisome proliferator-activated receptor γ (PPARγ) and CEBPs, and promote lipolysis by enhancing the activity and translocation of hormone-sensitive lipases in mature adipocytes [144]. On the contrary, Lee *et al* reported eosinophil-driven adipocyte maturation and lipid accumulation. In addition, IL4 treatment increased lipogenic gene expression including *Cebpe*, *Acaca*, *Fasn*, and *Scd*. In turn, adipocytes can support ATE migration and survival by producing IL3, IL5, and GM-CSF [74].

Another pathway linking ATE and thermogenesis/browning involves a newly identified signalling molecule meteorin-like hormone (METRNL). METRNL could be expressed by skeletal muscles and adipocytes upon cold exposure, promoting Th2 immune response and thermogenesis through ATE accumulation and ATM activation [89]. In human, METRNL could directly regulate adipocyte differentiation, and adipogenesis [145]. ATE could also produce METRNL, which is under the control of transcriptional repressor KLF3 [77]. Accordingly, KLF3-deficient mice had a smaller body size, lower adipose tissue content, increased heat production, and were protected from obesity [77]. This phenotype correlated with a drastic increase of ATE expressing *Metrnl* and *Il33* [77]. In addition, *Klf3*^−/−^ ATE promoted the thermogenic reprogramming of mature adipocytes *in vitro* [77]. Altogether, these data supported a pivotal role of ATE KLF/METRNL/IL33 axis in controlling AT thermogenesis.

In addition to cold exposure, catecholamines, such as NE, could also activate thermogenic programs of existing brown and beige adipocytes through β3-AR and induce *de novo* beige fat formation via β1-AR [146, 147]. Interestingly, eosinophils in perivascular AT (PVAT) were found to express TH, the rate-limiting enzyme in catecholamine biosynthesis, and catecholamines (epinephrine, norepinephrine, and dopamine) could be produced *in vitro* by ATE [148]. However, the effect of eosinophil-derived catecholamines in eWAT/scWAT during obesity/thermogenesis has not been assessed. Instead, the ATE-derived catecholamines acted on blood vessels inducing relaxation via the mechanism involving nitric oxide (NO) and adiponectin secreted by adipocytes [148]. Finally, blood vessels containing PVAT from ΔdblGATA mice were more constricted upon stimulation compared to those derived from WT animals (Figure 2).

### AT innervation

Catecholamines derived from the sympathetic nervous system (SNS) innervating AT are the most well-known circuit implicated in adaptive thermogenesis, WAT beiging, and lipolysis. AT is highly innervated, and its innervation depends on the AT metabolic state. Thus, obesity and its complications decreased AT SNS, whereas cold exposure, caloric deprivation, or leptin exposure promote its SNS innervation [147, 149, 150]. The sole activation of the SNS fibre in AT is sufficient to promote lipolysis and AT beiging. Interestingly, ATE were found to promote SNS axonal branching in AT via neuronal growth factor (NGF) production [151]. More precisely, Meng *et al* recently reported that upon cold exposure, SNS-derived NE orchestrate stromal cells to release IL33 which in turn activates ILC2. Subsequently, activated ILC2 secrete IL5, further recruiting ATE, which in turn produces NGF and allows SNS axon branching [151]. NGF producing eosinophils were also found in human [152], indicating that a similar pathway could occur in humans as well. While only correlative, the age-induced loss and dysregulation of ILC2 observed by Goldberg et *al* [60] could potentially induce the age-related AT denervation observed by others [153] and via a mechanism involving ATE. In agreement with the study from Meng *et al*, Haberman e*t al.* recently identified the AT stromal cell producing IL33 [112]. Indeed leptin-sensing LepR^+^ SAPC are the barrier surrounding sympathetic axon bundles in AT [112, 154], and upon stimulation they were able to produce IL33 [112]. Most importantly, the specific deletion of IL33 in SAPC induced a significant decrease of eosinophils in BAT, subsequently promoting BAT inflammation [112]. Thus, SAPC-derived IL33 were proposed to be the link between ILC2/ATE in AT homeostasis [56].

In addition to SNS, AT is endowed with less well-characterized somatosensorial innervation [155]. Interestingly, selective AT sensory nerve ablation enhanced both lipogenesis and thermogenesis, resulting in larger AT enriched in beige adipocytes and elevated thermogenesis [155]. On the other hand, eosinophils in lung were shown to be able to communicate with lung somatosensory innervation via molecules such as CCL11, ICAM1, VCAM1, and eosinophil peroxidase (for review [156]). As a result, the putative role of ATE in somatosensorial AT niche needs to be further explored.

## Role of eosinophils in obesity-associated complications

Obesity often results in complications such as NASH leading to more severe NAFLD, or even fibrosis and cirrhosis [5, 6]. Moreover, these inflammatory diseases are implicated in hepatocarcinogenesis and a strong link between obesity and liver cancers has been identified [7]. While the protective role of eosinophils in AT is well reported, their role on obesity-associated liver complications is scarce. Th2 immune response and IL4/IL13 have been reported to have a protective role after liver injury. Indeed, following acute liver injury, CCL11 is produced by fibroblast and epithelial in response to increased IL13 signalling, thereby promoting eosinophil infiltration in the injured liver. Subsequently, eosinophil-derived IL4 acted on IL4Rα^+^ hepatocytes to promote their proliferation, liver regeneration and healing [157]. More recently, using macrophage or IL33 deficient mice it was reported that eosinophil accumulation in injured liver depends on macrophage-derived CCL24 [158]. However, in addition to hepatocyte regeneration, the Th2 cytokine IL13/IL4 also promotes fibroblast collagen secretion, and an excessive activation of this pathway lead to fibrosis [159]. In addition, IL13 could collaborate with transforming grow factor β (TGFβ) thereby amplifying fibrosis, and IL13 was upregulated in NASH model [160]. Accordingly, blocking of both IL13/TGFβ result in greater benefice in NASH liver compared to blocking of TGFβ alone. These evidences support the hypothesis that Th2 immune response in liver promote fibrosis and NASH complication in HFD-induced obesity, in contrast to what was observed in AT [160]. Finally, in liver, eosinophil associated cytokines such as IL4, IL5, and IL13 were upregulated in NASH patients compared to healthy individuals, reinforcing the idea of detriment role of eosinophils in metabolic liver diseases in human [160].

Obesity is very often associated to T2D, a metabolic disease characterized by high concentrations of glucose in the blood, leading over time to damage in various organs [2, 3]. However, while the role of eosinophils in pancreatic disorders begun to be addressed [161], their role in T2D is completely lacking. Studies suggest a correlation of eosinophil counts with T2D induced nephropathy [162], while other report an inverse correlation of eosinophils and T2D [100]. Finally in cohort of patient infected by *Strongyloides stercoralis*, an increase of eosinophilia was observed, and this correlated with lower incident of T2D and associated metabolic disorders [101]. Thus, more studies are needed to understand the role of eosinophils in metabolic diseases.

## Concluding remarks

ATE are part of a complex and a well-coordinated network of cells implicated in maintenance of AT homeostasis. Their role in immunometabolism is just beginning to be explored, and many questions remain to be answered. Indeed, mice lacking eosinophils provide strong evidence about the role of ATE in AT homeostasis and disease. However contradictory data across study let the doubt install: Is ATE abundance alone important or is the stimuli promoting metabolically beneficial ATE? In addition, the AT depots across body are functionally different: Does each AT depot require a specific population of eosinophils for its homeostasis? Moreover, the importance of ATE versus ILC2 or ATM in AT beiging still remain unanswered. ATM is an heterogenous population of cells and the specific population coordinating ATE recruitment and maintain is still unknown. On the other hand, ATE heterogeneity is still not addressed: Do distinct subpopulations of ATE exist, as shown for other organs and what is their subtissular niche of residency?

Of note, the human studies cited in this review were not designed to target the role of eosinophils in metabolic disorders, nevertheless their data do not support the protective role described in animal studies. However, the difficulty to interpret these results come from the fact that patients with severe asthma are often on corticosteroid treatment and the disease sequel may limit their active lifestyle thereby leading to weight gain. Therefore, the weight loss observed may be due to reduced steroid treatment and/or increased of physical activity. In conclusion, studies focused on addressing the role of human eosinophils in metabolic disorders need to be further designed.

Overall, additional work is urgently needed in the quest to identify whether ATE are a heterogeneous population and if functionally different ATE across AT depot exists with AT depot-specific function.

## Data Availability

N/A

## Abbreviations

AP: adipocyte progenitors
AR: adrenergic receptor
ASC: adventitial stromal cells
AT: adipose tissue
ATE: adipose tissue eosinophils
ATM: adipose tissue macrophage
BAT: brown adipose tissue
BM: bone marrow
BMI: body mass index
C/EBP: CCAAT/enhancer-binding protein
CCR2: C-C chemokine receptor 2
CCR3: C-C chemokine receptor 3
CLS: crow-like structures
CMP: common myeloid progenitor
CSF2R: Colony Stimulating Factor 2 Receptor
EoP: eosinophil lineage-committed progenitor
eWAT: epididymal white adipose tissue
FGF21: fibroblast growth factor 21
GM-CSF: granulocyte-macrophage colony-stimulating factor
GMP: granulocyte/macrophage progenitor
HFD: high-fat diet
ICAM-1: intercellular adhesion molecule
IFN: interferon
IL: interleukin
ILC: innate lymphoid cells
IRF: IFN response factor
KLB: co-receptor β-Klotho
KLF3: Kruppel-like Factor 3
LepR: leptin receptor
METRNL: meteorin-like hormone
MSC: multipotent stromal cells
NAFLD: non-alcoholic fatty liver disease
NASH: non-alcoholic steatohepatitis
NE: norepinephrine
NGF: neuronal growth factor
NMUR1: Neuromedin U receptor 1
NO: nitric oxide
PD-L1: Programmed death-ligand 1
PDGFR: platelet-derived growth factor receptor
PPAR: peroxisome proliferator-activated receptor
PVAT: peri-vascular adipose tissue
SAPC: Sympathetic Associated Perineurial Cell
scRNAseq: single cell RNA sequencing
scWAT: subcutaneous white adipose tissue
SNS: sympathetic nervous system
STAT: Signal transducer and activator of transcription
SVF: stromal/vascular fraction
T2D: type 2 diabetes
TF: transcription factors
TGFβ: transforming grow factor β
TH: tyrosine hydroxylase
Th1: T helper type 1
Th2: T helper type 2
TNF: tumour necrosis factor
TSLP: thymic stromal lymphopoietinv
UCP1: uncoupling protein 1
VCAM-1: vascular cell adhesion molecule 1
WT: wild-type
XBP1: X-box-binding protein-1

## Permission to Reproduce

N/A

## Clinical Trial Registration

N/A
